# Inter-professional education and primary care: EFPC position paper

**DOI:** 10.1017/S1463423619000653

**Published:** 2019-10-04

**Authors:** Robin Miller, Nynke Scherpbier, Loes van Amsterdam, Virgínia Guedes, Peter Pype

**Affiliations:** 1 Head of Department of Social Work and Social Care and Co-Director of the Centre for Leadership in Health and Social Care, University of Birmingham, Birmingham, UK; 2 Head of Primary Care Specialty Training, Department of Primary and Community Care, Radboud University Medical Center, Nijmegen, the Netherlands; 3 Social and Organisational Scientist, Independent Consultancy, IJsselstein, the Netherlands; 4 Center for Health Technology and Services Research, Northern Regional Health Administration, Baixo-Tâmega Health Centers Grouping, Porto, Portugal; 5 Department of Public Health and Primary Care, Research Unit Interprofessional Collaboration in Education and Practice, Ghent University, Ghent, Belgium

**Keywords:** best practice, education, EFPC, inter-professional, primary care, professional

## Abstract

Inter-professional education (IPE) can support professionals in developing their ability to work collaboratively. This position paper from the European Forum for Primary Care considers the design and implementation of IPE within primary care. This paper is based on workshops and is an evidence review of good practice. Enablers of IPE programmes are involving patients in the design and delivery, providing a holistic focus, focussing on practical actions, deploying multi-modal learning formats and activities, including more than two professions, evaluating formative and summative aspects, and encouraging team-based working. Guidance for the successful implementation of IPE is set out with examples from qualifying and continuing professional development programmes.

## Introduction

The strength of primary care is that it engages a diverse and rich range of professionals, organisations, and sectors. While all of these have particular roles and associated knowledge and skills, there is increasing recognition of the need for professionals within primary care to better collaborate together (Samuelson *et al.*, 2012, Langins and Borgermans, 2015). This reflects the changing needs of populations throughout Europe and in particular the increase in those with multiple long-term conditions (European Union, 2017). It also builds on the expectation of patients and their families that professionals will communicate effectively in order to provide coordinated and person-centred support. However, many people continue to experience fragmented care in which professionals within primary care do not work together successfully (National Voices, 2012). Most health systems also struggle to ensure that there is good collaboration between primary and secondary care services, and between health and social care. Collectively, these fragmentations result in poorer outcomes for patients, the inefficient use of resources, and/or greater likelihood of failures in safety. A lack of understanding of the roles of others is a key contributor to such fragmented care (Samuelson *et al.*, 2012; Supper *et al.*, 2015). Primary care professionals do not automatically have the skills, knowledge, and values necessary to practice collaboratively with the range of professionals that they will engage with throughout their career (Xyrichis and Lowton, 2008; Mangan *et al.*, 2015). The uni-professional nature of the education and development that the most traditionally experienced does not properly prepare them for such practice (Frenk *et al.*, 2010).

Addressing such fragmentation and achieving the benefits of improved coordination requires intervention at all levels of the system (Valentijn *et al.*, 2015; Miller *et al.*, 2016). This includes supportive policy frameworks, motivating financial incentives, enabling organisational cultures, and well-led multi-professional teams (Samuelson *et al.*, 2012). Evidence further highlights that professionals having the right competences to work positively with other professionals are an important foundation of person-centred, goal-orientated, and coordinated care (Baker, 2010). These include communication with those of other professionals and disciplines, having clarity about the boundaries of own and other roles, putting patient and family at the centre of care, and working within teams including the ability to constructively resolve inter-professional conflicts (Canadian Interprofessional Health Collaborative, 2010; IPEC, 2016). Learning with other professionals can facilitate the development, consolidation, and enhancement of the skills, values, and behaviours necessary for more collaborative practice. Reviews of inter-professional education (IPE) in general report that learners respond well positively, their attitudes/perceptions of one another improve, and they gain knowledge and skills necessary for collaborative practice (eg, Reeves *et al.*, 2016; Mertens *et al.*, 2018). Impact on organisational practice and improvements in outcomes for patients and communities are less clear, although there is evidence that some programmes have made a positive difference.

In primary care, there are distinct issues relating to inter-professional working that differ from those within hospital-based settings run by large public or private sector bureaucracies (Samuelson *et al.*, 2012; McInnes *et al.*, 2015; OECD, 2019). These vary between national contexts, but commonly include employment within a variety of organisational structures, including small enterprises and professionally owned businesses; a diverse mix of professionals, including those from non-regulated groups and emerging roles; fluid teams that may not meet physically on a regular basis but work as virtual, dynamic networks; connection with other sectors outside health and social care, such as education, employment, housing, criminal justice; and multiple forms of integration, including ‘vertical’ integration with secondary care in hospital and ‘horizontal’ across primary care and wider community resources. This suggests that while generic approaches will have some relevance there is a need for IPE to understand and respond to the distinct context and challenges of primary care.

Developed by an inter-professional group from across Europe and at different career stages, this position paper aims to give an overview of the essentials of IPE in primary care. It is based on interactive workshops at the European Forum for Primary Care (EFPC) conferences attended by those from a range of professional and academic backgrounds, and a literature review of best practice examples in primary Care IPE (Box 2). Following a general overview of IPE in primary care, this paper sets out enablers to achieve good practice in IPE and how such programmes can be implemented successfully in primary care.

Box 1. Key definitions

*Professionals*. Core professionals within primary care include general practitioners and practice-based nurses, with other common professionals including community nurses, midwives, dentists, physiotherapists, social workers, psychologists, occupational therapists, speech therapists, dieticians, and pharmacists. In this paper, the term professional also includes those undertaking skilled roles within primary care that may not have a formal professional status. This would include, for examples, those in support roles, administrative staff, and managers.

*Multi-professional collaboration* describes situations in which professionals work alongside but in the most part independent of each other (Mahler *et al.*, 2014)

*Inter-professional collaboration* occurs when multiple health and care workers from different professional backgrounds work together to provide comprehensive services by working with patients, their families, carers and communities to deliver the highest quality of care across settings (WHO, 2010)

*IPE* occurs when students from two or more professions learn about, from and with each other to enable effective collaboration and improve health outcomes (WHO, 2015)

*Undergraduate education* is formal learning which that leads to a degree and a professional qualification and which is generally undertaken at university, college, or medical school

*Post-graduate education* is formal learning after the basic professional degree which leads to a higher degree and/or specialist qualification

*Continuing professional development* (CPD) is on-going learning throughout a primary care professional’s career though informal and formal learning opportunities.

Box 2. Literature review process

A literature search was undertaken by the specialist health library at the University of Birmingham in Healthcare Management Information Consortium (HMIC), Medline, Embase, Cinahl, and PubMed databases between May to July of 2018. It used the following search terms: inter-professional OR multi-professional OR interdisciplinary OR multidisciplinary OR interagency OR multiagency OR transdisciplinary OR collaborative (run in keyword field except in PubMed) AND education OR learning OR curriculum OR teaching OR development (run in title) AND ‘primary care’ OR ‘primary healthcare’ OR ‘family medicine’ OR ‘general practice’ AND 2000 present. In total, 622 articles were identified with an initial short-listing of articles through the reading of abstracts. Inclusion criteria were that studies related to undergraduate, post-graduate, or continuous development programs developed in primary care contexts which appeared to demonstrate positive outcomes in relation to the collaborative competence of the learners and/or impacts for patients and/or communities. Studies developed exclusively in other contexts, such as hospitals, were excluded. Shortlisted articles were read in detail by the researchers to confirm that they met the inclusion criteria. When the articles linked to other published research, snow balling to other articles was undertaken. A total of 24 articles were selected, and after a detailed reading, each one was summarised, completing a standard template that included the educational level, overview of IPE approach, the good practice aspects, how it was evaluated, and other comments. These were circulated around the research team with discussion and debate between its members leading to the identification of enablers of good practice.

## Overview of IPE within primary care

Primary care IPE should not be seen as an end in itself but introduced as a targeted means to improve outcomes for patients and the efficiency and effectiveness of health and care services. Educators should therefore be attuned to the wider practice, policy, social, and economic environment. They should seek to develop a good understanding of the particular challenges and/or opportunities that the IPE will contribute. These may be demonstrated on a *strategic* level in relation to an overall improvement in health systems (Mann *et al.*, 2009; Lennox and Anderson, 2012; Miller *et al.*, 2014), on a *community* level in relation to local inequalities (Larivaara and Taanila, 2004; Ryan *et al.*, 2015), on *team* level in relation to a particular service configuration (Rugen *et al.*, 2014), and/or in relation to individual *professional* practice level (Meisinger *et al*., 2016). To understand the potential contribution of IPE, it is worth considering barriers related to collaboration, for example, siloed working within health and social care, poor team working, hierarchical and physician-centred culture, and professional (rather than person)-centred emphasis within care (Zaudke *et al.*, 2016; Meisinger *et al*., 2016). Involving different agencies and professionals in the analysis of problem enables a fuller understanding to be developed and secures interest from these stakeholders if a new IPE programme is seen to have relevance. This initial assessment must understand what is important to patients, families, and communities. While this can build on previous research or feedback, it should ideally seek to involve them as active partners.

Having identified the relevant societal problems and collaboration challenges, it must be considered how IPE can contribute to achieving these desired outcomes. The goal of IPE could be to improve collaboration in a more general way through helping professionals to understand the contribution of each other and enhance working relations (Delva *et al.*, 2008). This commonly involves development of teamwork skills, appreciation of each profession’s scope of practice, and collaborative practice skills (Price *et al.*, 2009). Lennox and Anderson (2012) argue that in preparing the health and social care workforce for outcome-based practice, the development of technical skills should be complemented with skills for eﬀective team working and collaborative practice. IPE projects are often focussed on improving the health and well-being of specific patient groups whose care requires intervention and support from numerous professions and services and/or changes to lifestyle alongside clinical treatment. Examples of such IPE programmes include helping patients live with chronic back pain (Worswick *et al.*, 2015), self-managed diabetes (Paquette-Warren *et al.*, 2014), cope with the health and social impacts for them and their families of dementia (Lee *et al.*, 2013). The objectives can be more than professionals learning how to collaborate, for example, facilitating patient involvement in learning (Worswick *et al.*, 2015) or undertaking quality improvement projects (Paquette-Warren *et al.*, 2014).

Many teaching and learning methods are suitable for IPE. The design (and indeed delivery and evaluation) should be underpinned by relevant educational theories. Common theories deployed in IPE include those related to cognition, social constructivism, intergroup processes, and power (Hean *et al.*, 2018). Practice-based and practice-relevant education can be organised by discussing real patient cases during rotations in primary care practices (McNair *et al.*, 2005). Clinical placements can be combined with didactic courses on inter-professional collaboration and case studies (Mann *et al.*, 2009; Lennox and Anderson, 2012; Kent *et al.*, 2014; Sicat *et al.*, 2014) or individual portfolios, small group exercises including chart reviews, case presentations, and longitudinal quality improvement projects to learn and implement in the same curriculum (Singh *et al.*, 2005). Preceptors can be from the students’ own discipline (McNair *et al.*, 2005) or mixed (Singh *et al.*, 2005). An alternative to real case studies is that trained patient educators can be used for student interviews (Solomon, 2011; Towle and Godolphin, 2013). Observation of inter-professional teams on the workplace should be complemented with critical dialogue, reflections, and joint discussions between students and professionals (Barr *et al.*, 2017). The more authentic the experiences the more students will learn (Schrader *et al.*, 2016). Involving patients and family members as partners of an inter-professional team can demonstrate to learners that there is potential for new forms of professional–patient interactions (Crutcher *et al.*, 2004; Rugen *et al.*, 2014). Wider competences such as clinical leadership can be acquired through student-run inter-professional clinics (Meisinger *et al*., 2016).

CPD initiatives commonly involve workshops with individual and small group case-based exercises to discuss patient vignettes (Delva *et al.*, 2008; Balogh *et al.*, 2015), encounters with trained simulated patients (Davis *et al.*, 2008), or real cases from participants’ own practices (Kanisin-Overton *et al.*, 2009). Involving service users, patients, or their representatives in the workshops makes it even more practice relevant (Worswick *et al.*, 2015). Team-simulation exercises on patient case studies with group debriefing allow for a range of competences to be acquired (Strachan *et al.*, 2011). Multi-method modules involving active teaching, small group work, active sharing of experiences in a programme adapted to the expressed needs of the participants with feedback options after each part of the sessions allow for a range of knowledge, skills, and attitudes to be addressed (Hearnshaw *et al.*, 2001). Focusing on workplace learning in practice can be another efficient approach for CPD (Mertens *et al.*, 2018). At its most minimal, this can be discussing case scenarios in online modules with professionals from the same practice (Balogh *et al.*, 2015) and applying the learnt content in practice before moving to the next module (Moyer). A continuous practice-based learning effort with supervision can turn the team into a learning collaborative (Paquette-Warren *et al.*, 2014).

## Enablers of primary care IPE

The good practice examples of IPE within primary care identified common enablers within such programmes – involving patients in the design and delivery, providing a holistic focus, focussing on practical actions, deploying multi-modal learning formats and activities, including more than two professions, evaluating formative and summative aspects, and encouraging team-based working. Not all of these enablers were contained within all of the examples, and programmes involving a formal academic qualification more thoroughly demonstrated multiple themes than shorter and more focussed CPD programmes (see Tables 1–3). That should not be taken to imply that CPD programmes were not effective – rather that their aims were more limited due to a focus on a particular condition or social situation for which existing collaboration was ineffective and/or an innovation was available to improve outcomes. This differed from the broader range of underpinning competences sought by undergraduate and post-graduate programmes and which were more substantial in terms of resources and time. It is further worth noting that learning about collaboration can (and indeed should) be included within uni-professional programmes (ie, IPE is not the only relevant mode of learning). This can involve an inter-professional component through engaging faculty from different professional backgrounds within the curriculum. Similarly, while the focus of this paper is on IPE, there is also a need for disciplines within the same profession to understand how to better collaborate (Janssen *et al.*, 2017).

**Table 1.** Undergraduate good practice examples

Author	Date	Country	Involved patients	Holistic focus	Practical action	Multi-modal	Multi- professional	Team based	Robustly evaluated
Towle and Godolphin	2013	Canada	XX	XX			XX		
Mann *et al.*	2009	Canada	XX	XX	XX	XX	XX	XX	XX
Zaudke *et al.*	2016	USA		XX	XX	XX	XX	XX	XX
Kent *et al.*	2014	Australia	XX	XX	XX		XX	XX	
Solomon	2011	Canada	XX	XX			XX		
Sicat *et al.*	2014	USA	XX		XX	XX			
Ryan *et al.*	2015	USA	XX	XX	XX	XX	XX	XX	
McNair *et al.*	2005	Australia	XX	XX	XX	XX	XX	XX	XX
Lennox and Anderson	2012	UK	XX	XX	XX	XX	XX	XX	XX

**Table 2.** Post-graduate good practice examples

Author	Date	Country	Involved patients	Holistic focus	Practical action	Multi-modal	Multi-professional	Team based	Robustly evaluated
Singh *et al.*	2005	USA	XX		XX	XX	XX	XX	XX
Meisinger *et al.*	2016	USA	XX	XX	XX	XX	XX	XX	XX
Larivaara and Taanila	2004	Finland	XX	XX	XX	XX	XX	XX	XX
Price *et al.*	2009	Canada	XX	XX	XX	XX	XX	XX	XX
Rugen *et al*.	2014	USA	XX	XX	XX	XX	XX	XX	XX

Source: In some cases, these programmes also included undergraduate students

**Table 3.** CPD good practice examples

Author	Date	Country	Involved patients	Holistic focus	Practical action	Multi-modal	Multi- professional	Robustly evaluated	Team based
Worswick *et al*.	2015	UK	XX	XX	XX	XX	XX	XX	
Oeseburg *et al.*	2013	Netherlands		XX		XX			XX
Parekh *et al.*	2015	Australia				XX	XX		XX
Moyer	2016	USA			XX	XX	XX	XX	
Paquette-Warren *et al.*	2014	Canada			XX	XX	XX	XX	XX
Delva *et al.*	2008	Canada		XX	XX		XX		
Lee *et al.*	2013	Canada	XX		XX	XX	XX	XX	XX
Quinn *et al.*	2008	Australia	XX	XX			XX		XX
Curran *et al.*	2007	Canada				XX	XX		XX
Miller *et al.*	2014	UK		XX	XX	XX	XX	XX	XX

### Involving patients

The first-hand perspective of patients was used within many programmes to demonstrate to learners how real health and social needs do not fit neatly into the structures imposed by services and how poor coordination would impact their experience and outcomes. This could be through patients presenting their stories during a development session and/or through learners being asked to respond to the current needs of individuals and their families (Lennox and Anderson, 2012; Meisinger *et al*., 2016). Visiting a patient in their own home helped to reinforce the importance of personal context and for learners to engage with the patient as human being (Mann *et al.*, 2009; Zuadke *et al.*, 2016). Involving patients and communities from an early stage enabled them to shape the focus and aims of the programme. This was achieved through recruiting patient representatives to be members of a steering group and/or seeking the views of patients through surveys or other consultative processes (Kent *et al.*, 2014). While helpful, such involvement still favours professionals and educators as the experts. More radical approaches can further redress this power imbalance. For example, employing community educators to design and lead sessions based on their own experiences conveyed to learners the need for personalised care to respond to the diversity of individual need and situation (Towle and Godolphin, 2013), and including patients as ‘co-learners’ within an improvement programme changed the professional–patients dynamics to one in which ‘openness and equality became the norm’ (Worswick *et al.*, 2015: 286). Ensuring that patients are properly prepared, supported, and confident in undertaking their role is an important element of the design (Solomon, 2011) and can result in patients reporting personal benefits (Lennox and Anderson, 2012; Worswick *et al*., 2015).

### Holistic focus

Inter-professional working becomes necessary when people have more health and social care needs that can only be properly addressed through intervention and support by multiple professionals. This is often related to people having more than one condition and/or complex social needs, or successful management of their condition requiring lifestyle changes alongside clinical treatments. Learning accordingly benefits from developmental approaches that facilitate participants in gaining a more rounded view of the person/population, their situation, and potential resources (Mann *et al.*, 2009; Miller *et al.*, 2014). Many programmes involved learners from different professions undertaking assessment of a person in order to develop a shared understanding and integrated care plan (Price *et al.*, 2009; Kent *et al.*, 2014; Sicat *et al*., 2014; Meisinger *et al*., 2016). Patients sharing how their health issues had affected their lives similarly encouraged more integrated responses as this would start from the premise of what is important to them rather than clinical pathways (ie, ‘what matters?’ rather than ‘what is the matter’?) (Solomon, 2011; Lennox and Anderson, 2012; Towle and Godolphin, 2013). Exploring the social determinants of health and the contribution that communities could play in promoting well-being provides a further level of understanding (McNair *et al.*, 2005). Una Vida Sana involves participants being based within Hispanic communities to engage with the realities of exclusion and work with local people in responding to their challenges (Ryan *et al.*, 2015).

### Practically orientated

Opportunity for learners to reflect on their lived experience of working inter-professionally was central to many programmes. Practical activities were also a means to achieving wider impacts than improved competence of the participants. Supporting individual patients, for example, at a time of transition such as leaving hospital, enabled learners to experience many steps involved and secure a good discharge for the person and their family (Mann *et al.*, 2009; Kent *et al.*, 2014; Rugen *et al.*, 2014; Sicat *et al.*, 2014; Meisinger *et al*., 2016; Zaudke *et al.*, 2016). Reflecting on the insights of an individual and/or group of people was used by a number of programmes as the basis for students to identify practical ways that services and/or collaboration could be improved in that local area (McNair *et al.*, 2005; Delva *et al.*, 2008; Lennox and Anderson, 2012; Ryan *et al.*, 2015). Working on a partnership challenge faced by primary care and other agencies enabled participants in one CPD programme to develop a relevant business case and to learn more about how their colleagues’ organisations made decisions about resource investment (Miller *et al.*, 2014). Similarly, IPE supported local implementation of local memory clinics as well as opportunities for individual and shared learning (Lee *et al.*, 2013).

### Multi-modal

Multiple learning activities and modes of delivery help to consolidate learning and provide opportunities for those with different learning styles. These included in-person lectures, online learning platforms, group discussions, team tasks, individual and/or collective reflections, observations of clinics, community development, improvement projects, and others (Oeseburg *et al.*, 2013; Parekh *et al.*, 2015; Moyer, 2016). The staging of content and activities to provide opportunity for application and reflection is important (Paquette-Warren *et al.*, 2014). The Leicester model was designed around Kolb’s learning cycle with concrete experiences such as visiting a patient and interviewing professionals being used to encourage abstract thinking and then experimentation of potential solutions (Lennox and Anderson, 2012). While content is principally decided by faculty (in conjunction with a steering group), it can be helpful to also build in flexibility for participants to shape minor or major aspects of the curriculum (Larivaara and Taanila, 2004; Miller *et al.*, 2014). Considering how to integrate informal learning alongside, more formal sessions can help to support deeper understanding and connect theory to practice. Informal learning can be facilitated through connecting participants with other learners from different professions, observing and undertaking tasks with experienced practitioners. This includes students who were at a later stage of their degree as well as those who were professionally qualified and many years of service (Meisinger *et al*., 2016). Environmental enablers for interaction and relationship building included common team rooms and online discussion platforms (Price *et al.*, 2009).

### Multi-professional (and multi-agency)

According to the IPE principles, two or more professions use to be involved as learners in undergraduate, post-graduate, and CPD programs. Some of the good practice examples were limited to nurses and physicians or general practitioners (Oeseburg *et al.*, 2013; Rugen *et al.*, 2014; Meisinger *et al.*, 2016). Others also included pharmacists, social workers, occupational and/or physical therapists, dietitians, psychologists, dentists, administrators, or others (Delva *et al.*, 2008; Mann *et al.*, 2009; Price *et al.*, 2009; Solomon, 2011; Lennox and Anderson, 2012; Lee *et al.*, 2013; Towle and Godolphin, 2013; Miller *et al.*, 2014; Paquete-Warren *et al.*, 2014; Parekh *et al.*, 2015; Ryan *et al.*, 2015; Zaudke *et al.*, 2016). In some cases, programs included different disciplines within the same profession, as is the case of ‘Partnerships for Health’, a continuum development professional program dedicated to improving chronic care in primary care, which includes, in addition to other professionals, registered nurses, diabetes nurses educator, nurses practitioner, and health promoter registered practical nurses (Paquette-Warren *et al.*, 2014). Facilitators should also be multi-professional, involve academics and practitioners, and with an interest in delivering IPE. The faculty team may be from disciplines of family medicine, pharmacy, nursing, social work, geriatric medicine, safety engineering, behavioural science, occupational therapy, physiotherapy, health services and health information administrations, psychology, law, and others (Singh *et al.*, 2005; Lee *et al.*, 2013; Paquette-Warren *et al.*, 2014; Zaudke *et al.*, 2016). It could have participation of facilitators from different sites, such as hospital-based collaborative practice and community-based long-term care (Mann *et al.*, 2009). There is often a multi-agency steering group planning and overseeing the learning. This can involve universities schools and other agencies, such as health institutions, the social system, and community entities (Delva *et al.*, 2008; Mann *et al.*, 2009; Lennox and Anderson, 2012; Lee *et al.*, 2013; Miller *et al.*, 2013; Rugen *et al.*, 2014; Ryan *et al.*, 2015). For example, for the development of a patient-centred IPE intervention, in which the patient was the teacher, it was created an Advisory Group comprised the core project team, faculty, students, and representatives of community-based patient advocacy (Towle *et al.*, 2013).

### Team based

IPE programs are aimed to give learners the opportunity to engage with content in teams. Learners are involved in a group project with a specific purpose in which the program integrates. The Crimson Care Collaborative Clinic in Family Medicine teaches exclusively teams (medicine and nurse practitioner students), providing real patient care team experiences, which allows sharing their profession-specific knowledge and skills, builds trust and intends to soften the hierarchy between professions (Meisinger *et al.*, 2016). Team-based students’ activities can include the patient interview and the development of an integrative care plan, based on the information and resources from each one profession, as is the case in Seamless care. Furthermore, the team functioning was accessed by a Team Reflexive Exercise completed by the students following each team meeting (Mann *et al.*, 2009). The Leicester Model of IPE (Lennox and Anderson, 2012) is composed of teams of four students, to develop together an education cycle initiated with patients and workers’ interviews and finalised with the presentation of solutions of management of attendance of the team. In the same way, inter-professional teams from an existing service can attend the learning together. There can be related to quality improvement in which primary care teams develop a project to improve the chronic care (Lee *et al.*, 2013; Paquette-Warren *et al.*, 2014; Worswick *et al.*, 2015).

### Robustly evaluated

IPE programs evaluation considers impact as well as participant experience that must include patients, learners, faculty, and preceptors. Prior and post surveys can be applied (Lennox and Anderson, 2012; Lee *et al.*, 2013; Miller *et al*., 2014; Parekh *et al.*, 2015; Ryan *et al.*, 2015; Zaudke *et al*., 2016). Analysis includes qualitative and quantitative methods, depending on the research question and the resources available. Quantitative data collecting can include scales, such as The Role Performance Questionnaire (Skinner *et al.*, 2005), the Students Attitudes Toward Community Service (Wall *et al.*, 2007), the Readiness for Inter-professional Learning Scale (Mattick *et al.*, 2009), and the Interdisciplinary Education Perception Scale (McFadyen *et al.*, 2007). Qualitative data can be collected by log books, handouts, reports, observation, and interviews (Paquete-Warren *et al.*, 2014). Price *et al.* (2009) developed a guide based on the concepts of success IPE in the literature to guide a semi-structural interview. Other authors applied semi-structured interview (Lennox and Anderson, 2012; Miller *et al.*, 2013), including the patients (Mann *et al.*, 2009). Focus group was also used in learners (Mann *et al.*, 2009; Miller *et al.*, 2014) and preceptors (Mann *et al.*, 2009) such as open-ended write questions to increase assessment (Lennox and Anderson, 2012; Miller *et al.*, 2013; Oeseburg *et al.*, 2013). Kirkpatrick’s model of educational outcomes was used by several authors (Mann *et al.*, 2009; Lee *et al.*, 2013; Miller *et al.*, 2014) to present the findings of the program evaluation, demonstrating the outcome levels of reaction, learn-modification of attitudes and perceptions, acquisition of knowledge and skills, behaviour and changes to organisational practice and benefits to clients. Participants’ expectations regard to the program and advices to improve are taken into account (Oeseburg *et al.*, 2013), and all evaluation is used to improve subsequent iterations of the programme.

## Implementation of IPE within primary care

There are particular challenges when introducing a new IPE programme within primary care. This will often involve contributions from and collaboration between the educational faculty who may not usually work together. It can be seen as an additional demand to the core professional curriculums with associated complexities regarding timetabling and access to core resources such as rooms and technology. CPD programmes are usually trying to secure the interest of busy primary care professionals who will also be trying to juggle demands from their clinical work and home life. It is therefore vital that the practicalities of implementation are considered at an early stage.

A core planning group, which engages the range of educators relating to the professions involved in the curriculum, should consider the logistical issues that will need to be addressed. This can be advised and challenged by a steering group consisting of relevant external stakeholders (Mayall *et al.*, 2004). They can also help to connect with other agencies and gain their practical support. For example, CPD programmes can be more sustainable and impactful when delivered within practices with specialist educators providing oversight and guidance (Lee *et al.*, 2013; Paquette-Warren *et al.*, 2014), and protected time in practice will enhance participation in IPE CPD (Strachan *et al.*, 2011). Establishing a collaboration between educational institutions and clinical practices allows for co-creation and continuous refinement of the programme (Price *et al.*, 2009; Meisinger *et al*., 2016). Engagement with senior leadership both at the institutional level and at the level of each individual professional programme is necessary to secure their commitment, which is needed in decisions on curriculum change, human resources, and budget. Practical arrangements that support IPE development include protected time for those leading the curriculum (Mayall *et al.*, 2004), administrative support with overall coordination and timetabling, office space, equipment, and technological support (Pottie *et al.*, 2009 Paquette-Warren *et al.*, 2014). A key factor for success is ensuring that there are development opportunities for faculty to ensure that they are confident in relation to the content and approach of the curriculum (Singh *et al.*, 2005). In addition, more general opportunities for educators to learn with those from other professional programmes help to socialise them into general role models of IPE (Barr *et al*., 2017; Lawlis *et al.*, 2014; Brashers *et al.*, 2016; Schrader *et al.*, 2016). External funding helps to provide capacity for staff to undertake the curriculum development and implementation (McNair *et al.*, 2005; Curran *et al*., 2007; Mann *et al.*, 2009; Price *et al*., 2009; Oeseburg *et al.*, 2013; Towle and Godolphin, 2013; Worswick *et al.*, 2015; Moyer, 2016). The aim is be to integrate professional and inter-professional competencies longitudinally rather than creating separate modules.

Using the model of Kern (2016), the steps below suggest key issues to be considered in the development and implementation of a new IPE programme in primary care. These are illustrated through practical examples.

*Step 1: What are the societal issues (ie, health, well-being, inequality, and/or economics) which are of concern and what is the contribution of inter-professional collaboration in primary care to achieve the ideal outcomes?*

- Professional, policy, academic, and other data should be drawn upon to understand the need for more collaborative working.
- The need assessment should outline what ‘ideal’ outcomes would be for the population concerned and then compare this with what is achieved in practice.
- The views of the patients and communities concerned should be gained in respect of the ideal scenario and identifying gaps.
- The potential contribution of inter-professional collaboration (ie, between which professionals, at what point, in which context) in achieving these should be established.

### Good practice example: Una Vida Sana (Cox *et al.*, 2014; Ryan *et al.*, 2015)

The Latino population in the USA is recognised as facing many barriers to accessing health care services due to language differences, limited transportation, and a lack of awareness of how the system works. Working with the Schools of Pharmacy and Nursing, medical faculty members at the Virginia Commonwealth University met with Latino community members and faith-based organisations who work with Latino families to identify local barriers to health and consider how an IPE project could positively impact. Other partners involved in the development included the Office of Multicultural Affairs within the municipal authority, a large free-to-access health clinic, and an interpreter service within the University. This resulted in the learning activities focussing on mixed professional groups of students organising and delivering community-based health education events.

- *Step 2: How well do existing educational arrangements support collaboration in this area of primary care practice?*
- An analysis of educational arrangements for the professionals concerned is undertaken to understand how these support (or not) the necessary collaboration.
- Education across the spectrum should be considered.
- This analysis should be undertaken by a team of educators and professionals that reflects the diversity of professions and services involved in the area of collaboration.
- The most relevant career stages of the professionals concerned are considered.

### Good practice example: Primary Care Medical Homes (Moyer, 2016)

Team-based holistic working within primary care practices in central Pennsylvania was introduced over a five-year period. While this led to greater consistency of specific activities such as setting goals with patients and preventative testing, focus groups with practice staff discovered that many did not understand the broader concept of the Primary Care Medical Home (PCMH) and what benefits this could provide for patients and the community. A multi-professional executive team reviewed the competence of the staff within primary care to deliver the new model. The review found that existing training had neither enabled staff to engage with the theoretical underpinnings of the PCMH nor prepared them to be more patient-led in their practice and to work constructively with other professions. To address these issues, they introduced an inter-professional programme in which practice members from different professions would work together with patient representatives to undertake a quality improvement project.

*Step 3: What are the aims and objectives to be achieved through IPE within primary care?*

- The overall aims and specific learning objectives of the IPE programme should be set to address the identified needs.
- The aims should be holistic and consider the quadruple aim – learner experience, patient outcomes, population health and inequalities, and efficient use of resources.
- Perspectives of patients and communities should influence the setting of aims and objectives.
- Programmes should be aspirational but also feasible in relation to resources and timescales.
- Aims and objectives may vary for the various professionals involved but should be made explicit for each professional group.
- Evaluation methods and supporting data sources should be considered.

### Good practice example: rural healthcare (McNair *et al.*, 2005)

In line with most countries, rural areas in Australia can struggle to attract sufficient health professionals to meet the needs of their communities. A traditional tendency of universities to be more connected with urban settings is thought to contribute to this workforce challenge. The Rural Inter-Professional Education project therefore sought to encourage future professionals to consider working in rural areas alongside those preparing for different roles. Its learning objectives included effective consultation with community agencies in a rural setting to understand the resources available and to enhance interest in learners of gaining further experience in rural health care. In addition to these general aims, the students worked in teams to undertake a project that responded to a local need and that was developed in consultation with the relevant community.

*Step 4: What are the IPE educational strategies and methods that will support these learning aims and objectives to be achieved and are additional resources required/available?*

- IPE can draw on diverse teaching approaches dependent on the objectives, learners, and contexts.
- Group- and active-based learning provide opportunity to directly experience professional collaboration.
- Directly working with patients and families (in inter-professional groupings) highlights the real-life challenges and opportunities of collaboration.
- Observation of professional practice and experiences in the workplace should be completed by critical reflections between students and professionals.

### Good practice example: continuing education to improve chronic care (Paquette-Warren *et al.*, 2014)

The Partnerships for Health Project in Southwest Ontario aimed to improve the care of people with diabetes through an inter-professional, quality-improved-based education programme. Learning centred on practice teams (which also had to include a community-based healthcare profession) undertaking quality improvement activities. Formal educational input on underpinning topics such as inter-professional care, improvement techniques, and diabetes guidance was provided in their home practice or off-site. This was complemented by more facilitative development activities such as monthly teleconferences to review progress, reflect on material, and provide further expert input, and coaches meeting regularly with teams to encourage reflection and connect with other practices. There were also additional materials available through web-based platforms. The flexibility, and range of learning opportunities within the programme, was seen by participants as important to ensure that it was relevant to their local context.

*Step 5: How will the IPE curriculum be implemented and potential barriers overcome in primary care and educational environments?*

- Core planning team reflecting the professional faculties and key stakeholders (eg, professional or community placements) should coordinate implementation.
- Engagement of senior leadership within educational institution and support functions such as HR and Finance help to secure endorsement and necessary resources.
- Faculty may themselves need development and support to deliver IPE and work with learners from different backgrounds.
- Education to develop uni-professional and inter-professional competencies should be integrated rather than seen as parallel processes.

### Good practice example: the Leicester model (Lennox and Anderson, 2012)

Based on the Kolb cycle of experiential learning, the Leicester Model of Inter-professional Learning brings together students to work in small groups in a primary care setting. These inter-professional groups interview a patient in order to understand their personal priorities, the impact of broader societal issues, and their direct experience of health and care services. Students then compare the perspective of patients with that of professionals currently providing support. Insights regarding opportunities to improve people’s care are then presented back to local agencies. The approach has been successfully used with undergraduate and post-graduate students and has been extended to settings outside of primary care. To support its development and implementation, there has been continued engagement with students, the wider university, and community stakeholders. This has helped to secure continuous interest and to resolve potential practical difficulties such as timetabling and integration with the general curriculum (Lennox and Petersen, 1998). When an adapted version was being introduced for students training to work with people with a disability, the educators undertook a series of actions to help with the implementation (Anderson *et al.*, 2010). This included meeting with senior managers from local agencies to gain their perspective and secure their support, ensuring that the planned community sites had sufficient capacity, and using away days to engage tutors with the content and inter-professional focus.

*Step 6: How will the programme be evaluated to assess summative impact and provide formative learning for future primary care IPE?*

- Evaluation approach should be agreed before the programme is implemented to ensure early learning of process and robust measurement of outcomes.
- Mixed methods can strengthen evaluation and in particular if they accommodate validated quantitative tools along with qualitative data.
- Sharing findings with stakeholders encourages on-going interest and can facilitate discussion of how programmes can be improved.
- Publishing findings helps to share learning about what worked and what could be improved for future IPE programmes.

### Good practice example: strategic change programme (Miller *et al.*, 2014)

The Integrated Care Development Programme (ICDP) was a continuing inter-professional educational programme for senior primary care professionals, managers, and funders. Strategic teams from a single locality participated in university and workplace-based learning activities centred on the development of an integrated business plan to address a local priority for improvement. The ICDP had three aims – to increase the efficiency of health and care services, to increase the competency of staff in collaboration, and to develop partnerships between agencies and services. The evaluation sought not only to assess participant experience and self-reported learning, but also impacts on their behaviour and organisational effectiveness. Data were gathered before, during, and after the programme. Mixed methods were used to explore different aspects of the expected process and outcomes. These included use of the Interdisciplinary Education Perception Scale (McFadyen *et al.*, 2007); end of teaching block ratings regarding organisation, learning, interest, and pace of sessions, with qualitative comments to explain ratings; individual (survey) and collective (group) reflections on programme design, context, and overall learning; and semi-structured interviews six months post-programme regarding application of learning and progress in implementing business case. Focus groups were also held with the core faculty to gain their insights on the process of design and delivery.

## Conclusion

Inter-professional collaboration, which is crucial for primary care, is to meet the needs of populations that are ageing and with multiple chronic conditions in a way that is person-centred, effective, and sustainable (Samuelson *et al.*, 2012). IPE has an important role to play in professionals developing the competences required to collaborate successfully. It should complement uni-professional education exploring collaboration to ensure that learners are able to build their understanding and develop confidence in their abilities to work with others. IPE opportunities can be beneficial throughout professional careers in both qualifying and CPD programmes and deployed effectively within the implementation of quality improvement and other practical initiatives. While the focus of this paper has been on professions connected with the direct delivery of primary care services, there is also much potential in drawing in a wider range of disciplines and professionals to understanding and responding to the complex and changing needs of individuals and population. While much is known about how to implement IPE, there is also still much to be learnt. It is therefore important that robust research and learning-based evaluations of IPE programmes are undertaken and shared. The European Forum for Primary Care is committed to the benefits of IPE and providing useful guidance and supportive challenge to our practice, policy, and academic communities.
